# Clinical characteristics of anterior cerebral artery (ACA) territory infarction caused by congenital absence of bilateral ACA: a case report

**DOI:** 10.1007/s13760-020-01534-9

**Published:** 2020-11-06

**Authors:** Fei Guo, Liming Cao, Lijie Ren

**Affiliations:** 1 Department of Neurology, Huazhong University of Science and Technology Union Shenzhen Hospital (Nanshan Hospital), Shenzhen, China; 2 Department of Neurology, The 3Rd Affiliated Hospital of Shenzhen University, 47 Friendship Road, Luohu District, Shenzhen, 518000 China; 3 Department of Neurology, Shenzhen University First Affiliated Hospital, Shenzhen, China; 4 Department of Neurology, Shenzhen Second People’s Hospital, Shenzhen, China

**Keywords:** Anterior cerebral artery, Brain infarction, Stroke, Congenital abnormalities, Intracranial arteriovenous malformations

Dear Editor,

Cerebral infarcts in the anterior cerebral artery (ACA) territory are relatively rare, representing 0.5–3% of all ischemic strokes. Arteriosclerosis is a common cause of cerebral infarctions in the ACA territory [1] and occasionally leads to cardiogenic embolism [2]. However, other research suggests that arterial dissection (AD) is the most common cause of isolated ACA territory infarctions [3]. The incidence of unilateral A1 segment aplasia, three A2 segments, unpaired A2 segment, and fenestrations of the A1 and/or A2 segment is 5.6%, 3.0%, 2.0%, and 1.2%, respectively [4]. Recurrent ACA territory infarctions caused by the bilateral absence of the complete ACA are rarely reported. Therefore, the clinical characteristics of recurrent infarctions in the ACA territory due to this anatomical variation remain unknown.

In July 2019, a 76-year-old man presented with a 6-h history of left limb weakness. He had hypertension 6 months previously and no history of major trauma, toxic exposure, or hereditary disease. Physical examination at admission showed a blood pressure of 151/92 mmHg, regular heart rhythm, clear consciousness, motor aphasia, shallow nasolabial sulcus on the left side, distortion of commissure, and decreased muscle strength in the upper (0/5) and lower (4/5) left limbs. His National Institutes of Health Stroke Score (NIHSS) was 6. Routine blood tests, fasting blood glucose level, blood electrolytes, and measures of liver, kidney, and thyroid functions were normal. Homocysteine (18.5 mmol/L) and triglyceride (3 mmol/L) levels were higher than normal, while his high-density lipoprotein (0.69 mmol/L) level was below the normal level. Tests for syphilis, human immunodeficiency virus, anti-myeloperoxidase, anti-glomerular basement membrane, and anti-proteinase three antibodies were negative. Computed tomography (CT) showed multiple lacunar cerebral infarctions in the right frontal lobe. Echocardiography showed decreased left ventricular diastolic function. Magnetic resonance (MR) imaging (MRI) showed multiple, chronic (Fig. 1a) and acute (Fig. 1b) infarctions in the right frontal lobe and an old, watershed infarct in the right junction of the temporal and occipital lobes (Fig. 1c). Brain MR angiography (MRA) showed the bilateral absence of the ACA and local stenosis in the M2 segment of the right middle (MCA) and posterior (PCA) cerebral artery (Fig. 1d). Cerebral digital subtraction angiography (DSA) showed bilateral absence of the ACA (Fig. 1e, f, h), and the PCA (Fig. 1g) and MCA (Fig. 1i–j) were compensating for blood supply insufficiency in the ACA. Acetylsalicylic acid (100 mg/day), clopidogrel bisulfate (75 mg/day), atorvastatin calcium (20 mg/day), and butylphthalide (50 mg/day) were administered. His symptoms improved significantly, and he was discharged after 8 days, once his NIHSS and Modified Rankin Scale score were 1. At the 6-months follow-up, his NIHSS was 0.

**Fig. 1:**
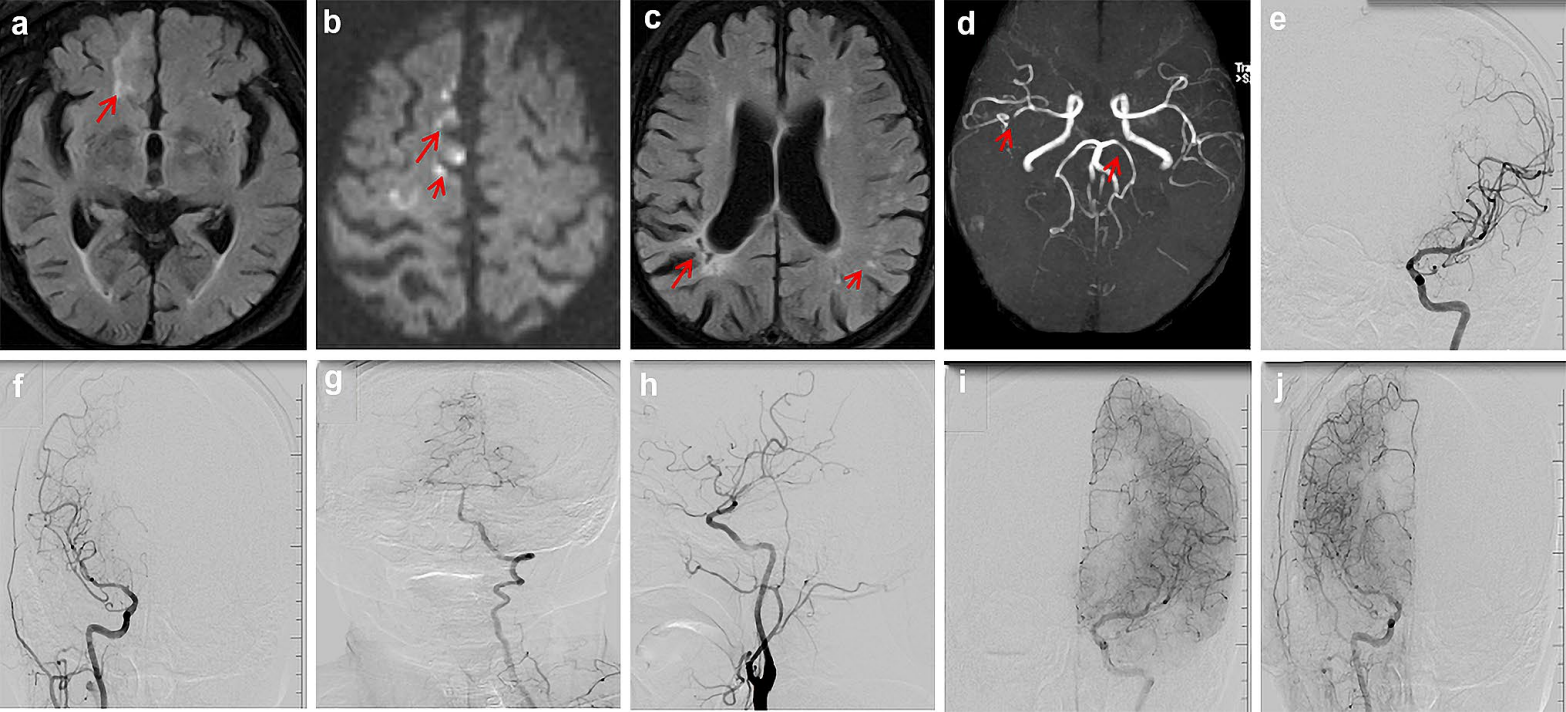
**a**, **b** Flair sequence and diffusion-weighted imaging show old ischemic white matter lesions (**a**, arrow) and multiple acute punctate infarctions (**b**, arrows), respectively, in the right frontal lobe. **c** Flair sequence shows an old watershed infarct in the right junction of the temporal and occipital lobes (long arrow) and multiple left-sided subcortical punctate ischemic demyelinating lesions (short arrow). **d** Brain MRA shows the bilateral absence of the ACA, right MCA M2 segment mild stenosis (arrow), and left PCA P1 segment mild stenosis (arrow). **e**, **f**, **g** Frontal DSA shows the absence of the left (**e**) and right (**f**) ACAs, and the PCA compensates for the ACA through the pial artery in the late arterial phase (**g**). **h** Lateral DSA shows the absence of the right ACA. **i**, **j** DSA shows that the left MCA compensates for the left ACA through the pial artery (**i**), while the right MCA compensates for the right ACA through the pial artery (**j**). *ACA* anterior cerebral artery, *CT* computed tomography, *DSA* digital subtraction angiography, *Flair* fluid attenuated inversion recovery, *MRA* magnetic resonance angiography, *MCA* middle cerebral artery, *PCA* posterior cerebral artery

Bilateral absence of the ACA is a rare cause of ACA territory infarction. This patient showed recurrent ischemic foci in the ACA region. Additionally, the patient had a watershed infarction in the right junction of the temporal and occipital lobes, possibly associated with cerebral circulation decompensation and microembolism formation.

MRA showed the bilateral absence of the ACA A1 segments, although a stump was observed between the ends of the internal carotid artery and MCA. DSA excluded the possibility of an ACA occlusion vascular stump. Rather, the MCA and PCA compensated for blood supply insufficiency. The multiple acute infarctions, old infarctions and watershed infarction may have been associated with low perfusion caused by the absent ACAs. Furthermore, mild stenosis of the right MCA and left PCA also increased cerebral ischemia.

Atherosclerotic vessels frequently occur with artery-to-artery embolism, local branch occlusion by plaque, and *in* situ thrombosis, with the latter considered the most prevalent in ACA infarction [5]. AD is a significant mechanism of ACA stroke; however, it has only been observed in younger Japanese patients [6]. The topographic lesion patterns and consequent clinical features of ACA infarctions are determined based on diverse pathogenic mechanisms and collateral circulation status. Frontal lobe and corpus callosum dysfunctions are major features of ACA territory infarction [2], while incontinence and gait apraxia are two of the most common symptoms. This patient showed motor aphasia, which was presumedly caused by lesions in the dominant posterior part of the gyrus frontalis inferior (Broca’s area). As in this patient, a frontal lobe infarction causes contralateral limb dyskinesia, including mild hemiplegia and central facial and tongue palsy. Frontal lobe infarctions can also cause frontal mental disorders and cognitive dysfunction. Treatment of ACA territory infarction is similar to other infarctions and includes controlling risk factors, preventing complications, providing antiplatelet agents, administering hypolipidemic medications, improving cerebral circulation, and providing rehabilitation treatment. Intravenous thrombolytic therapy should be administered within the 4.5 h time window.

In conclusion, the absence of the complete ACA bilaterally is a rare cause of ACA territory infarction. This patient had recurrent ischemic foci in the ACA territory and a watershed infarction in the right junction of the temporal and occipital lobes. Physicians should pay attention to such presentations because timely treatment is crucial for recovery. Multicenter, comprehensive studies are needed in the future to confirm the clinical characteristics of such infarctions.

## Data Availability

Data sharing not applicable to this article as no datasets were generated or analyzed during the current study.
